# Beckwith-Wiedemann syndrome mimicking the classical form of congenital adrenal hyperplasia in newborn screening

**DOI:** 10.20945/2359-4292-2022-0395

**Published:** 2024-02-29

**Authors:** Jéssica Mallmann Erbes Schaefer Martins, Barbara Leitao Braga, Klevia Nunes Feitosa Sampaio, Tamires de Souza Garcia, Juliana Van de Sande Lee, Edson Cechinel, Genoir Simoni, Marilza Leal Nascimento, Paulo Cesar Alves da Silva, Maria C. V. Fragoso, Tania A. A. S. Bachega, Mirian Y. Nishi, Berenice B. Mendonca

**Affiliations:** 1 Hospital Infantil Joana de Gusmão Florianópolis SC Brasil Hospital Infantil Joana de Gusmão, Florianópolis, SC, Brasil; 2 Faculdade de Medicina da Universidade de São Paulo Hospital das Clínicas Departamento de Endocrinologia do Desenvolvimento São Paulo SP Brasil Departamento de Endocrinologia do Desenvolvimento, Hospital das Clínicas, Faculdade de Medicina da Universidade de São Paulo, São Paulo, SP, Brasil; 3 Universidade Federal de Santa Catarina Florianópolis SC Brasil Universidade Federal de Santa Catarina, Florianópolis, SC, Brasil

## Abstract

Beckwith-Wiedemann syndrome (BWS) is a common genetic congenital disease characterized by somatic overgrowth and its broad clinical spectrum includes pre- and post-natal macrosomia, macroglossia, visceromegaly, increased risk of neonatal hypoglycemia, and development of embryonic tumors. BWS occurs due to genetic/epigenetic changes involving growth-regulating genes, located on region 11p15, with an important genotype-phenotype correlation. Congenital adrenal hyperplasia (CAH) comprises a spectrum of autosomal recessive diseases presenting a variety of clinical manifestations due to a deficiency in one of the enzymes involved in cortisol secretion. Early diagnosis based on newborn screening prevents the adrenal crisis and early infant death. However, high 17-hydroxyprogesterone (17-OHP) levels can occur in newborns or premature infants without CAH, in situations of stress due to maternal or neonatal factors. Here, we report new cases of false-positive diagnosis of 21-hydroxylase deficiency during newborn screening – two girls and one boy with BWS. Methylation-specific multiplex ligation-dependent probe amplification revealed a gain of methylation in the H19 differentially methylated region. Notably, all three cases showed a complete normalization of biochemical changes, highlighting the transient nature of these hormonal findings that imitate the classical form of CAH. This report sheds light on a new cause of false-positive 21-hydroxylase deficiency diagnosis during newborn screening: Beckwith-Wiedemann syndrome.

## INTRODUCTION

Beckwith-Wiedemann syndrome (BWS) is a common genetic congenital disease characterized by somatic overgrowth, with an approximate incidence of 1 in 10,000-13,700 births. BWS broad clinical spectrum includes pre- and post-natal macrosomia, macroglossia, visceromegaly, increased risk of neonatal hypoglycemia, and development of embryonic tumors (1,2). The adrenal gland is frequently involved and may present diffuse cytomegaly of adrenal cortex, as well as cysts, adenomas, cortical carcinomas, neuroblastomas, and pheochromocytomas (3).

BWS is due to genetic/epigenetic changes involving growth-regulating genes, located on region 11p15, with an important genotype-phenotype correlation and about 80% of the patients having a known molecular defect on this region (4). Due to both varying molecular defects involving chromosome 11p15 and tissue mosaicism, patients can present with a variety of clinical features, leading to the newly defined BWS spectrum (BWSp). The BWSp can be further divided into three subsets of patients: those presenting with classic features, those presenting with isolated lateralized overgrowth (ILO), and those not fitting into the previous two categories, termed atypical BWSp (2). Mackay and cols. (5) suggested that the decision on a first-line test depends on the clinical phenotype of the patient, consensus guidelines and national regulations. The study suggested that single-locus testing may be the preferred option for a typical BWS phenotype, while multi-locus testing may be more appropriate for features that are characteristic of more than one imprinting disorder.

There are two imprinting regulators regions (ICRs) on chromosome 11, that influence important fetal growth genes. The telomeric region is called ICR1 and the centromeric region, ICR2. Each region regulates its specific domain: ICR1 is responsible for paternal expression of *IGF2* and maternal *H19*, and ICR2 is responsible for maternal expression of *CDKN1C* and paternal expression of *KCNQ1OT1* (6,7). These genes have monoallelic parent-of-origin dependent expression and failure in their regulation may result in abnormal gene expression during the prenatal period, causing opposite pathologies depending on the methylation profile of that region (8). For example, with the ICR1 gain of methylation (ICR1 GOM) on the maternal allele, patients with Silver-Russell syndrome have an overexpression of *H19*, which regulates the cell proliferation by suppressing it. However, in patients with BWS, the ICR1 GOM occurs in 5% to 10% of cases and the overexpressed gene is *IGF2,* which is involved in development and growth, directly corroborating with the features of the syndromes (5,6,9). Moreover, ICR2 loss of methylation (ICR2 LOM) on the maternal allele occurs in approximately 50% of the patients with BWS (5).

The most common test used for molecular diagnosis of BW and SR syndromes is methylation-specific multiplex ligation-dependent probe amplification (MS-MLPA). This molecular screening evaluates the number of copies (CNVs), quantifies the methylation of the target genes, and identifies uniparental disomy, including the possibility to semi-quantitatively profile methylation for multiple targets simultaneously (10).

Congenital adrenal hyperplasia (CAH) comprises a spectrum of autosomal recessive diseases presenting a variety of clinical manifestations due to a deficiency in one of the enzymes or proteins involved in the cortisol secretion (11). An elevated blood level of 17-hydroxyprogesterone (17-OHP) indicates 21-hydroxylase deficiency, and is used in neonatal screening of CAH. Early diagnosis based on newborn screening prevents the adrenal crisis and early infant death. However, high 17-OHP levels can occur in newborns or premature infants without CAH, in situations of stress due to maternal or neonatal factors (12). Elevation of 17-OHP can also occur due to adrenocortical tumors (ACT), which can lead to the neonatal diagnosis of these neoplasms (13).

Here, we report a new cause of false-positive 21-hydroxylase deficiency diagnosis in newborn screening identified in two girls and one boy: the Beckwith-Wiedemann syndrome.

## MATERIALS AND METHODS

### Case 1

A full-term female (39 weeks) born by an uneventful cesarean section with birth weight of 5.575 kg and birth length of 52 cm, was identified as a large for gestational age baby. Her mother denied any medication use or known illnesses during the pregnancy, including those related to fetal macrosomia, including gestational diabetes mellitus. She was born healthy with no perinatal complications, without occurrence of neonatal hypoglycemia or dehydration. However, she was referred to the Endocrinology service at the Clinical Hospital of the University of Sao Paulo due to abnormal neonatal screening results (17-OHP: 96 ng/mL) collected at 6 days old (reference value (RV) < 15 ng/mL).

She presented with normal female genitalia, ruling out the diagnosis of classical form of 21-hydroxylase deficiency. At 14 days old, she was 6.3 kg (Z: + 5.59), and 58 cm (Z: + 2.47), body mass index (BMI): 18.7 kg/m^2^ (Z: + 4.45), and presented syndromic characteristics as macroglossia, ogival palate, orbital hypertelorism, hepatomegaly, and umbilical hernia. Abdominal echography showed hepatomegaly, bilateral renal tumors, measuring 2 cm in the larger diameter and no adrenal tumors.

At one month and 14 days old, 17-OHP value was 7.4 ng/mL (RV < 15 ng/mL), androstenedione: 6.1 ng/mL (RV < 2.20 ng/mL), total testosterone: 279 ng/dL (RV < 48 ng/dL), 11-deoxycortisol: 2.11 (RV < 0.50 ng/mL). At five months old, values of 17OHP, androstenedione and testosterone were normal, but dehydroepiandrosterone sulphate (DHEAS) value remained high: 2913 ng/mL (VR < 1240 ng/mL). At 11 months old, DHEAS also normalized.

The patient was diagnosed with bilateral Wilms tumor at 11 months old and underwent chemotherapy with partial response requiring a left nephrectomy at the age of 1 year old (Table 1).

**Table 1:** Patient 1 from the Clinical Hospital of the University of Sao Paulo

Date	29/01/18	06/02/18	07/03/18	22/04/18	20/06/18	09/01/19
Age	6 days	14 days	1.5 month	3 months	5 months	11 months
LH (<0.15 U/L)	-	-	-	-	<0.15	<0.15
FSH (1-5 U/L)	-	-	-	-	5.0	3.9
17-OHP	96	7.1	7.4	1.36	1.36	<0.50
	(RV < 15 ng/mL)	(RV < 15 ng/mL)	(RV < 1.73 ng/mL)	(RV < 1.73 ng/mL)	(RV < 1.73 ng/mL)	(RV < 1.3 ng/mL)
Total testosterone (<48 ng/dL)	-	-	279	138	37	<12
Androstenedione (<2.2 ng/mL)	-	19.41	6.1	6.08	<0.50	<0.50
11-deoxicortisol (<0.50 ng/mL)	-	4.41	2.1	-	-	<0.50
DHEA-S (<1,240 ng/mL)	-	-	-	-	2913	81
Cortisol (6.7-22.6 µg/dL)	-	9.6	5.0	-	12.3	7.5
ACTH (7.2-63.3 pg/mL)	-	-	-	41.3	54.2	20.2

LH: luteinizing hormone; FSH: follicle stimulating hormone; 17-OHP: 17-hydroxyprogesterone; DHEA-S: dehydroepiandrosterone sulphate; ACTH: adrenocorticotropic hormone; RV: reference value.

### Case 2

A preterm male (34 weeks 4 days), large for gestational age (weight 3,670 g), was born by cesarean section due to breech presentation, after a healthy pregnancy. He presented macrosomia, macroglossia, umbilical hernia and auricular pits, with a clinical diagnosis of BWS.

He was referred to pediatric endocrinology service at Joana de Gusmão Children's Hospital for presenting high 17-OHP value in newborn screening: 70 ng/mL in first sample and 66 ng/mL in second sample collected (RV < 15 ng/mL), at 3 and 16 days, respectively. CAH investigation disclosed high basal testosterone levels (637 ng/mL), normal cortisol levels (11.4 mcg/L, RV 6.70 to 22.60 µg/dL), normal electrolytes and DHEAS > 10,000 ng/mL (RV < 1,240 ng/mL). Due to the high levels of DHEAS, an investigation of ACT was performed. Abdominal computed tomography (CT) showed bilateral enlarged adrenal glands, without tumors.

The clinical follow-up eight months later showed complete resolution of laboratory findings: 17-OHP 0.88 ng/mL (RV < 1.73 ng/mL), DHEAS 370 ng/mL (RV < 1,240 ng/mL), testosterone 13 ng/mL (RV < 48 ng/dL) (Table 2). Simultaneously, a new abdominal magnetic resonance imaging (MRI) showed no adrenal lesions, but identified a small nodular lesion with high cellularity in the diffusion sequence, in the posterior margin of the left kidney.

**Table 2:** Patient 2 from Joana de Gusmão Children's Hospital

Date	01/02/19	14/02/19	23/02/19	07/03/19	08/03/19	04/04/19	18/10/19	19/10/19	02/02/20
Age	3 days	16 days	25 days	1 month	1 month	2 months	8.5 months	8.5 months	1 year
Cortisol (6.7-22.6 µg/dL)	-	-	7.9	11.4	-	10.5	-	-	-
Plasma cortisol after dexamethasone suppression test (<1.8 µg/dL)	-	-	-	-	2.8	-	-	1.2	-
ACTH (7.2-63.3 pg/mL)	-	-	-	77	26	-	34	-	-
17-OHP	70.15 (RV < 15 ng/mL)	66.3 (RV < 15 ng/mL)	-	35.5 (RV < 4.72 ng/mL)	-	13.5 (RV < 4.72 ng/mL)	0.88 (RV < 1.73 ng/mL)	-	-
Total testosterone( 48 ng/dL)	-	-	637	299	-	393	13	-	-
Androstenedione (<2.2 ng/mL)	-	-	>10	-	-	>10	<0.3	-	-
DHEA-S (<1,240 ng/mL)	-	-	>10,000	>10,000	-	8,980	370	-	110

17-OHP: 17-hydroxyprogesterone; DHEA-S: dehydroepiandrosterone sulphate; ACTH: adrenocorticotropic hormone; RV: reference value.

At one year and two months old, another abdominal MRI showed an increase in renal nodular lesion, compatible with Wilms tumor. Chemotherapy treatment was initiated, and at one year and five months old, the child was admitted for left nephrectomy.

### Case 3

A preterm female (36 weeks), large for gestational age (4,295 g of weight, 51 cm length), and born by vaginal delivery. Her mother had gestational diabetes, but no pharmacological treatment was required. At 33 weeks of gestation, she underwent a preterm labor and received corticosteroid therapy.

After 48 hours of life, the baby began exhibiting episodes of hypoglycemia and was administered intravenous glucose for 3 days. Hypoglycemia did not reoccur after suspension of intravenous glucose. Post discharge, blood glucose levels remained normal with breast milk supply.

The baby was referred to the pediatric endocrinology service at Joana de Gusmão Children's Hospital at 29 days old for presenting a high 17-OHP value in newborn screening: 45 ng/mL (RV < 15 ng/mL). On physical examination, she had slight clitoromegaly, with adequate weight gain, without signs of dehydration. Laboratory tests and adrenal ultrasound were performed and a nonspecific small lesion in the right adrenal gland was found. DHEAS was > 10,000 ng/mL (RV < 1,240 ng/mL) and cortisol value was 8.28 µg/dL (RV 6.7 to 22.6 µg/dL).

Another adrenal ultrasound and abdominal CT showed a decrease of the adrenal lesion which was compatible with regression of adrenal haemorrhage.

Outpatient follow-up was performed with imaging and laboratory tests. Adrenal lesion showed a progressive reduction and at four months of age the adrenal ultrasound was normal. Gradual decrease in adrenal androgens was observed with complete normalization at nine months of age (Table 3).

**Table 3:** Patient 3 from Joana de Gusmão Children's Hospital

Data	14/04/19	05/05/19	09/05/19	20/05/19	21/05/19	06/06/19	15/08/19	10/02/20
Age	4 days	25 days	29 days	1 month	1 month	1.5 month	4.5 months	9 months
Cortisol (6.7-22.6 µg/dL)		-	-	-	-	10.9	-	8.2
Plasma cortisol after dexamethasone suppression test (<1.8 µg/dL)		-	-	-	1.4	-	-	-
ACTH (7.2-63.3 pg/mL)		36	49		5.4	-	-	-
17-OHP	44.97 (RV < 15 ng/mL)	>20 (RV < 15 ng/mL)	>20 (RV < 15 ng/mL)	9.2 (RV < 4.72 ng/mL)	-	12.8 (RV < 4.72 ng/mL)	4.1 (RV < 4.72 ng/mL)	-
Total Testosterone(<48 ng/dL)		-	197	105	-	108	38	<10
Androstenedione (<2.2 ng/mL)		-	>10	8	-	>10	6.7	<0.3
DHEA-S (<1,240 ng/mL)		-	>10,000	-	-	8,190	2,340	60

17-OHP: 17-hydroxyprogesterone; DHEA-S: dehydroepiandrosterone sulphate; ACTH: adrenocorticotropic hormone; RV: reference value.

## MOLECULAR DIAGNOSIS

**Figure 1:**
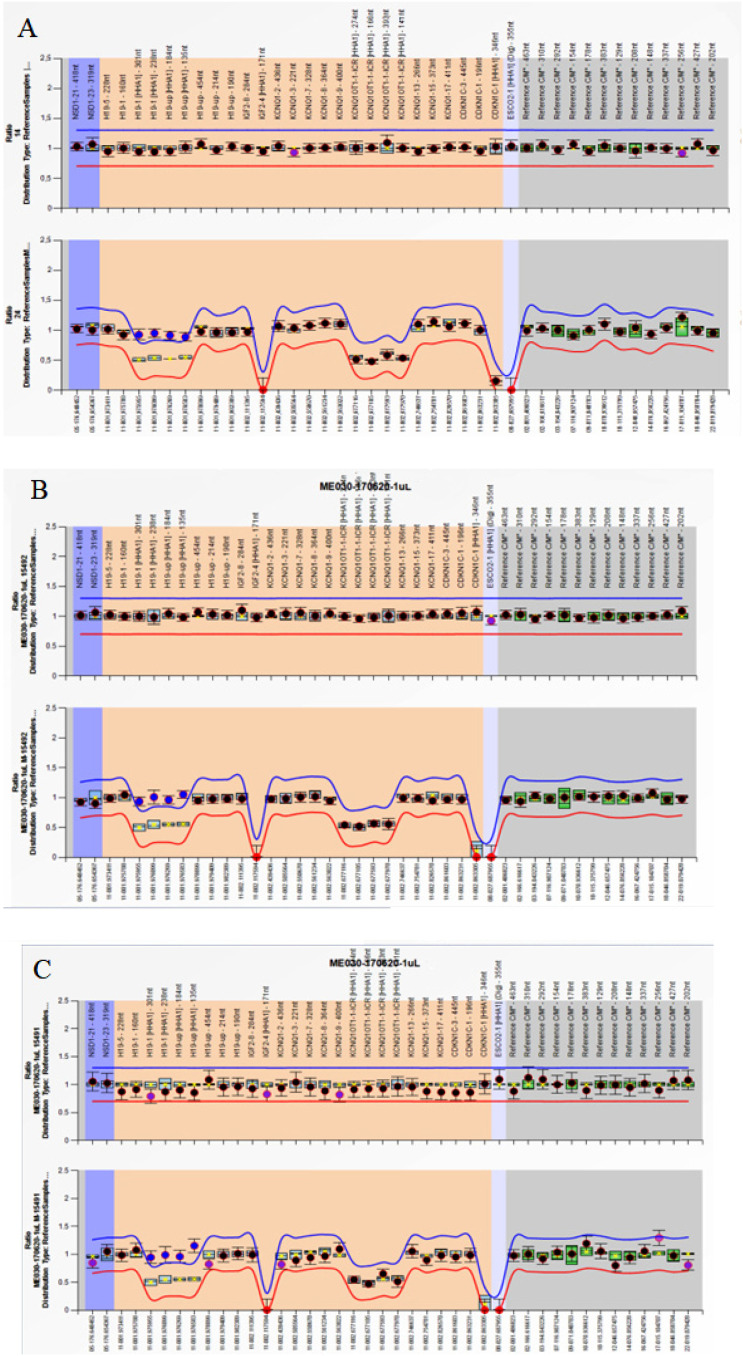
MS-MLPA results for Beckwith-Wiedemann syndrome. Probe ratios are indicated by dots. Blue and red lines are the 95% confidence interval (CI) of the reference samples. Black dots: within the 95% CI. Blue dots: outside the 95% CI. A, B, and C show normal copy number variation (CNV) and a gain of methylation (GOM) at ICR1 as the signal of the four probes for ICR1 increase to approach one. (**A**) Patient 1. (**B**) Patient 2. (**C**) Patient 3.

This study was approved by the local medical ethics committee and guardians gave their informed written consent. The study was approved by Plataforma Brasil at the reference number 35589720.1.0000.5361. Considering the clinical characteristics of our patients, MS-MLPA specific for SR and BW syndromes (SALSA MS-MLPA ME030 BWS/RSS) (MRC-Holland, Amsterdam, Netherlands) was selected as a first-line test, used for molecular diagnosis in these children. The molecular test was performed on blood samples, following the manufacturer's instruction, and showed a gain of methylation in the *H19* differentially methylated region, known as ICR1 of chromosome 11p15, which controls two imprinted genes, *H19* and insulin-like growth factor 2 (*IGF-2*), confirming the clinical diagnosis of BWS in these three children (Figure 1).

## DISCUSSION

CAH is a major cause of morbidity and mortality in children with the possibility of early diagnosis and effective treatment. Therefore, CAH has been included in the neonatal screening program worldwide that measures 17-OHP using a dried capillary blood collected on a filter paper. CAH newborn screening is efficient in detecting classical patients and can prevent neonatal mortality in children with the salt-wasting form, as well as prevent incorrect gender assignments in females. However, it is important to recognize other possible factors associated with increased 17-OHP levels in newborns without a diagnosis of 21-hydroxylase deficiency. The false-positive results create diagnostic difficulties, with therapeutic implications, especially in preterm newborns. For this reason, neonates with positive screening results should undergo additional tests to confirm the diagnosis (14). Adrenocortical tumors are part of the differential diagnosis of 17-OHP elevation, and individuals with BWS are at an increased risk for developing several tumors, including ACT (15,17).

The paternal uniparental disomy of chromosome 11 (upd(11p15)pat), which causes overexpression of *IGF2* and *KCNQ1OT1*, and loss of *H19* and *CDKN1C* expression, is responsible for approximately 20% of BWS cases, whereas the hypermethylation of ICR1, resulting in overexpression of *IGF2* and lack of *H19,* is found in 5% to 10% of the cases. In addition, mutations or deletions of imprinted genes on chromosome 11 and hypomethylation of the imprinting control region ICR2 are also responsible for BWS (6). Molecular abnormalities of the telomeric domain such as ICR1 GOM (found in our three patients) and upd(11p15)pat are associated with a major risk of embryonal tumors compared to the defects of the centromeric domain, for example ICR2 LOM and *CDKN1C* mutations (18). Two of our patients were diagnosed with Wilms tumor, which is consistent with the high risk for this tumor associated with hypermethylation of ICR1 (9,18,19). The concordance between molecular diagnosis and clinical features shows the importance of molecular screening for determining appropriate care and surveillance protocols (20), which has already been demonstrated in a study involving 407 individuals with BWS (18).

As a public hospital linked to a university, with limited resources to carry out complementary tests, we decided to stop the molecular investigation of additional imprinting sites, since the diagnosis was already confirmed, and focus on treatment and management of the patients’ clinical features.

Postnatal overexpression of *IGF2,* which is present in adults and children with ACT (20,22)*,* is also associated with adrenocortical hyperplasia and enhanced steroidogenesis in mice (23,26). In the presented cases, ACT was not diagnosed, but excessive androgen production was detected in the newborns. Bilateral adrenal hyperplasia may be present in BWS, even as a cause of Cushing syndrome (27), this could result from the delay of fetal adrenal gland maturation. Anatomopathological findings in BWS revealed massive adrenal enlargement due to a combination of cytomegaly, persistence of the transient cortex, and hyperplasia of the permanent cortex (27). The transient cortex produces large amounts of DHEA, which could explain elevation in serum DHEAS similar to ACT.

We presented the first description of false-positive diagnosis of classical CAH in newborn screening of three patients with BWS, probably due to persistence of transient adrenal cortex or overactivation of the permanent adrenal cortex. Due to the high risk of tumors in BWS, follow-up requires careful monitoring, and the molecular diagnosis is important for determining appropriate care and surveillance protocols.
